# Biophysical Evaluation of Rhesus Macaque Fc Gamma Receptors Reveals Similar IgG Fc Glycoform Preferences to Human Receptors

**DOI:** 10.3389/fimmu.2021.754710

**Published:** 2021-10-12

**Authors:** Andrew R. Crowley, Nana Yaw Osei-Owusu, Gillian Dekkers, Wenda Gao, Manfred Wuhrer, Diogo M. Magnani, Keith A. Reimann, Seth H. Pincus, Gestur Vidarsson, Margaret E. Ackerman

**Affiliations:** ^1^ Department of Microbiology and Immunology, Geisel School of Medicine at Dartmouth, Dartmouth College, Hanover, NH, United States; ^2^ Sanquin Research and Landsteiner Laboratory, Academic Medical Centre, Department of Experimental Immunohematology, University of Amsterdam, Amsterdam, Netherlands; ^3^ Antagen Pharmaceuticals Inc., Boston, MA, United States; ^4^ Center for Proteomics and Metabolomics, Leiden University Medical Center, Leiden, Netherlands; ^5^ Nonhuman Primate Reagent Resource, MassBiologics of the University of Massachusetts Medical School, Boston, MA, United States; ^6^ Department of Microbiology, Immunology and Parasitology, Louisiana State University Health Sciences Center, New Orleans, LA, United States; ^7^ Department of Chemistry and Biochemistry, Montana State University, Bozeman, MT, United States; ^8^ Thayer School of Engineering, Dartmouth College, Hanover, NH, United States

**Keywords:** nonhuman primate, IgG, Fc gamma receptor, N glycan, rhesus macaque, ADCC - antibody dependent cellular cytotoxicity, phagocytosis, complement dependent cytotoxicity

## Abstract

Rhesus macaques are a common non-human primate model used in the evaluation of human monoclonal antibodies, molecules whose effector functions depend on a conserved N-linked glycan in the Fc region. This carbohydrate is a target of glycoengineering efforts aimed at altering antibody effector function by modulating the affinity of Fcγ receptors. For example, a reduction in the overall core fucose content is one such strategy that can increase antibody-mediated cellular cytotoxicity by increasing Fc-FcγRIIIa affinity. While the position of the Fc glycan is conserved in macaques, differences in the frequency of glycoforms and the use of an alternate monosaccharide in sialylated glycan species add a degree of uncertainty to the testing of glycoengineered human antibodies in rhesus macaques. Using a panel of 16 human IgG1 glycovariants, we measured the affinities of macaque FcγRs for differing glycoforms *via* surface plasmon resonance. Our results suggest that macaques are a tractable species in which to test the effects of antibody glycoengineering.

## Introduction

Like many proteins destined for secretion, human immunoglobulin G (IgG) is subject to a variety of post-translational modifications as it migrates through the secretory pathway of plasma cells. A prominent modification is the attachment of an N-linked glycan to both heavy chains in the crystallizable fragment (Fc) portion (1). While the amino acid sequon is conserved, the precise identity of the specific N-linked glycoform incorporated is not genetically encoded. Among glycosylated IgG Fc domains, there exist a variety of observed glycoforms – 36 in total for humans (2), eight of which account for 90% of all IgG in normal sera (3). The frequency of IgG Fc glycoforms within the distribution of the total glycan repertoire tends to be predictable in healthy individuals (4), with some variation due to factors such as age, sex, and pregnancy (5–11). In general, this profile consists of high levels of fucosylation (95%), low levels of bisecting GlcNAc (15%), intermediate levels of galactose (45%), and low sialylation (10%) (3). This balance can be perturbed by a heightened immune response however (12, 13), and distinct antigen-specific antibody fractions may differ from each other and from the average serum IgG Fc glycan profile within a given individual (14–16).

Occupancy of this conserved glycosylation site is critical to the ability of an IgG molecule to interact with human Fcγ receptors (FcγR), as the absence or removal of the N-glycan produces an antibody with dramatically diminished or outright eliminated affinity for FcγR and no detectable effector function (17, 18). Slight changes in the composition of the Fc glycan can impart changes to the Fc-FcγR dynamic that may resonate all the way to the severity of clinical presentation (19–27). More specifically, an increase in galactose content has been associated with increased propensity of the Fc domain to hexamerize (28), as well as with a slight (≤2 fold) increase in affinity for most of the low affinity (i.e., not FcγRI) FcγRs (29, 30), while the absence of a fucose molecule branching from the asparagine-proximal N-acetylglucosamine (GlcNAc) has been credited with up to an astounding 50-fold increase in affinity for FcγRIIIa/b (31–33). This increase in affinity translates to improvement in antibody-dependent cellular cytotoxicity by FcγRIIIa-bearing natural killer (NK) cells (34, 35), which has made it an attractive tool for enhancing the efficacy of therapeutic monoclonal antibodies (mAbs) (36, 37).

Pre-clinical animal models serve as an important bridge for such glycoengineered mAbs migrating from the lab to the clinic. Like that of humans, macaque IgG features a conserved N-linked glycan motif, which is necessary for binding to macaque FcγR (38, 39). Despite having a degree of homology to humans that makes them a tractable and popular animal model for biomedical research, like all models, macaques can be sufficiently immunologically distinct that care should be exercised when attempting to extrapolate observations in non-human primates to humans (40). Whereas the macaque IgG subclasses are more functionally monolithic than those in humans (39), genetic diversity among FcγR is significantly greater among macaques than in humans, particularly for FcγRII (41). Additionally, macaques do not express an equivalent of the GPI-linked human FcγRIIIb, and the clinically-relevant functional differences in high and low FcγRIIIa binding affinity allotypes observed in humans are not reflected among frequent alleles in macaques (42). These characteristics help to set expectations and guide design and interpretation of experiments conducted in these models. Yet, other aspects of antibody immunobiology have yet to be fully investigated; the impact of antibody glycosylation on receptor binding is one such area of concern. Macaque IgG is more likely to feature a bisecting GlcNAc residue and shows greater variability in galactose content than human IgG Fc (43, 44). Furthermore, human IgG Fc sialylation is carried out with N-acetylneuraminic acid (NANA) whereas macaques use N-glycolylneuraminic acid (NGNA) (43). The most striking effect of glycovariation in human IgG is very clearly the “fucose effect” for FcγRIII, which has been proposed to be mediated by disruption of glycan-glycan contacts (45, 46) or *via* altered conformational sampling (47). Regardless of mechanism, this effect is readily observed from mice to humans (48), and the receptor glycosylation site associated with it is conserved in rhesus macaques (38). The available data suggest that the effect of afucosylation on macaque FcγRIIIa affinity is also conserved (49), although perhaps with more modest fold-change increases in affinity than what has been observed in humans.

Using a panel of glycoengineered human IgG1 antibodies, we report the affinities of the low affinity rhesus macaque (RM) Fcγ receptors for each of 16 glycoforms, and further validate our observations by analysis of relationships between rhesus serum IgG Fc glycan profiles and FcγR binding levels. This work provides a more complete picture of the glycopreferences of macaque FcγRs, allowing for more confident design of experiments and interpretation of data when engineered human antibodies are tested in non-human primate models.

## Results and Discussion

A panel of 16 previously described IgG Fc glycovariants generated *via* a series of chemical and genetic methods that alter the dominant species of glycan within production runs of an anti-trinitrophenol (TNP) human IgG1 monoclonal antibody (mAb) (50, 51) was used to investigate the glycopreferences of rhesus macaque FcγR recognition (**Supplemental Table 1**). Briefly, these tools selectively achieved a greater than nine-fold reduction in the amount of core fucose (from 95% to 10%), a ten-fold or greater increase in the frequency of bisected species (from 5% to >50%), and terminal sialylation (3% to >40%), as previously reported in greater detail (51). Variation in galactose content ranged from 10% to 80%. Collectively, this panel of variants represents well the diversity of glycan changes that exist within human serum and in recombinant expressed and glycoengineering monoclonal antibodies.

**Figure 1:**
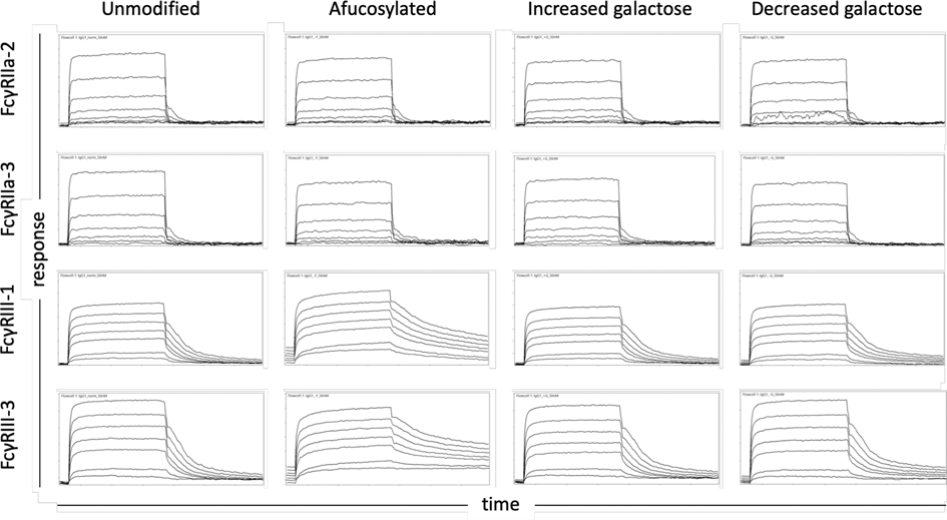
Exemplary sensor data of FcγR-IgG interactions. Sensorgams depicting the association and dissociation of FcγR from IgG over time for prevalent FcγRII (2 and 3) and FcγRIII (1 and 3) allotypes (rows) and differentially glycosylated IgG (columns). The equilibrium dissociation constants reported in this work were calculated using the response measured at the end of the association phase when interactions had achieved steady state. Each receptor was evaluated over a three order of magnitude concentration range.

The affinity of the low affinity macaque FcγRs for these variable glycoforms was measured using a multiplexed surface plasmon resonance (SPR) approach in which the glycovariants were covalently linked to a sensor chip and the FcγRs were the analyte in solution. Some of the differences imparted by modulating the Fc glycan were readily apparent from raw sensorgrams (**Figure 1**). While all the interactions exhibit a fast-on association dynamic, the off-rate was noticeably slower in the case of the higher affinity FcγRIIIa. This observation was particularly striking for interactions with afucosylated IgG wherein dissociation was not always complete by the end of the 5-minute step.

**Figure 2:**
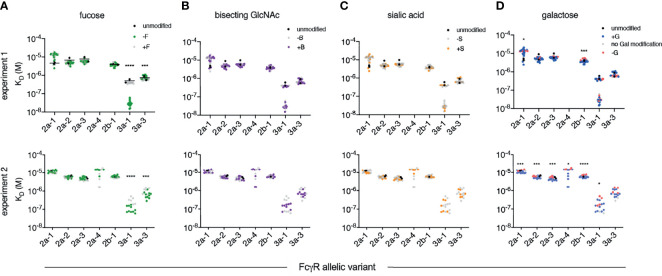
Affinity of rhesus macaque FcγRs for a panel of human IgG1 glycovariants. Equilibrium dissociation constants (K_D_) of rhesus macaque alleles having unique extracellular domains observed in two independent experiments (rows). Within each experiment, glycovariants were printed in replicate and each replicate is plotted. Results are presented such that each panel emphasizes a different category of glycomodification, including variable fucosylation (F) **(A)**, bisecting N-Acetylglucosamine (B) **(B)**, sialylation (S) **(C)**, and galactosylation (G) **(D)**. A natively glycosylated preparation (black) is plotted along with variants with (+) or lacking (-) the glycan emphasized in that panel. Statistically significant differences were tested using an unpaired t test comparing glycovariants with (+) and without (-) the modification (*p < 0.05, ***p < 0.0005, ****p < 0.0001).

**Figure 3:**
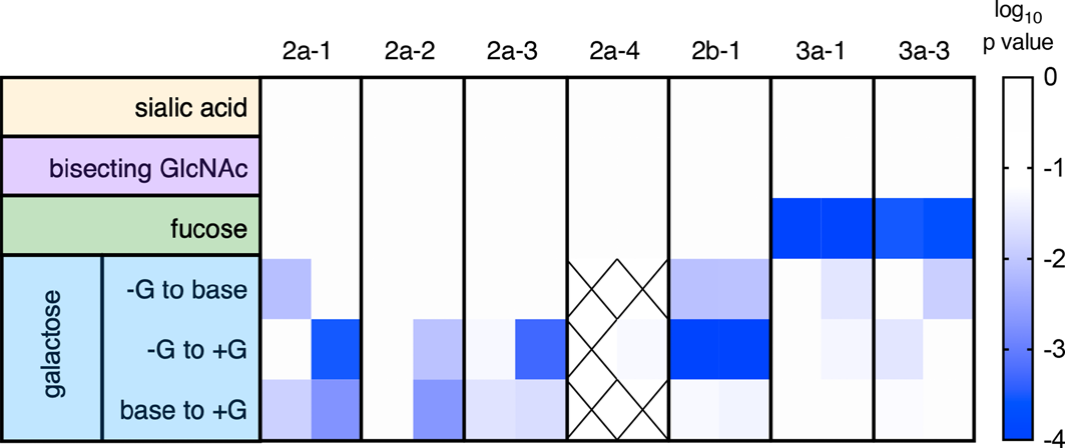
Summary of receptor affinity differences. Statistical significance of differences in affinity associated with variable glycosylation. Glycan modifications are tabulated by row and receptors by column, for each of the two independent experimental runs. Confidence in differences is indicated in color. Crosshatches indicate missing data. The effect of varying (+ *versus* -) sialic acid, bisecting GlcNAc, and fucose were evaluated by unpaired t test, and for galactose [+G, unmodified (base), and -G] with an ordinary one-way ANOVA corrected for multiple comparisons.

Equilibrium dissociation constants (K_D_) were fitted to the responses at steady state to define binding affinities for each receptor and IgG Fc glycoform variant in two separate experiments that compared the set of glycovariants with each modification to the set without (**Figure 2**). Among glycan modifications, reduction in core fucose of human IgG1 resulted in the most dramatic change in receptor binding affinity — improving the affinity of rhesus macaque FcγRIII as compared to Fc glycoforms without intentionally reduced fucose content (**Figure 2A**). While there was some variability in the magnitude and statistical confidence in the effect of reduced fucose between experimental runs and across the major FcγR allotypes (**Figure 3**), these results were generally consistent with a prior report of the sensitivity of RM FcγRIIIa to IgG Fc fucosylation (49).

In contrast, the presence or absence of bisecting GlcNAc did not have a statistically significant effect on the binding affinity of any RM FcγR tested (**Figures 2B**, **3**). While a prior study suggested that the presence of bisecting GlcNAc resulted in improved effector function in the context of human FcγR (52), other work has suggested that these observations were instead driven by variable fucosylation (51, 53). The observation that bisected glycans cannot subsequently become fucosylated appears to have resulted in some confounding of cause and effect with respect to the role of bisection (54).

Nor was there an impact associated with variable terminal sialic acid content (**Figures 2C**, **3**). Again, while some studies have suggested that sialic acid influences receptor binding affinity (55, 56), others have reported contradictory results (30, 57). Using this same well-characterized panel of glycovariants, variation in sialic acid content between 12-64% appeared to have essentially no general effect on binding affinity across diverse human FcγR (51).

**Figure 4:**
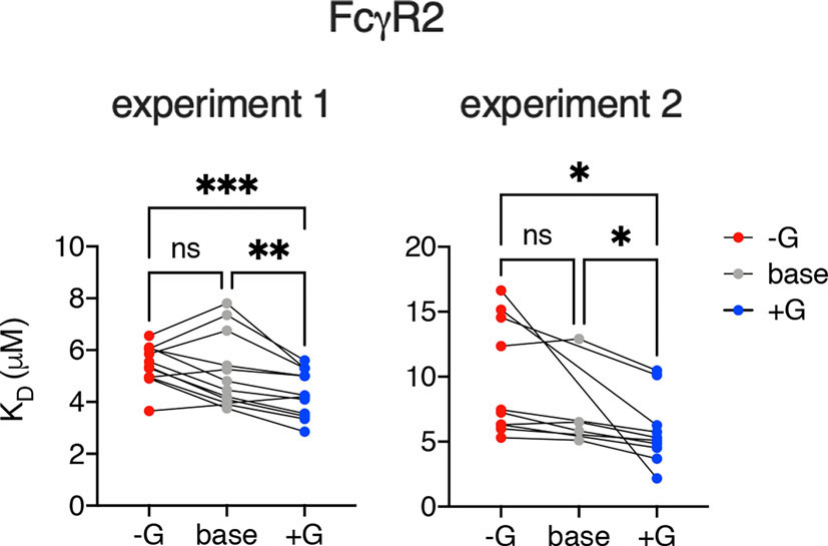
Increased galactosylation is associated with improved affinity for FcγRII. The effect of increased and decreased galactose content on binding to FcγRII variants among IgG Fc glycotypes for which other glycan characteristics (e.g., fucosylation, sialylation, bisection) were held constant. Statistically significant differences were tested using a mixed effect model with Tukey’s test of multiple comparisons (*p < 0.05, **p < 0.01, ***p < 0.001). ns, not significant.

Further mirroring their human counterparts, rhesus macaque FcγRs had marginally, but often statistically significantly, heightened affinities for IgG with increased levels of galactosylation (**Figures 2D**, **3**). To address these differences with improved resolution, analysis of the effect of variable galactosylation when fucose, sialic acid, and bisecting GlcNAc were held constant were analyzed in paired comparisons (**Figure 4**). As in the global data analysis, both experimental data sets showed a small but reproducible improvement to FcγRII binding affinity with increasing galactose content when other glycan attributes such as extent of fucosylation or bisection were held constant. These results are consistent with the phenotype observed in humans, where increasing galactose content improved affinity in a small but consistent manner among the low affinity FcγRs (30, 51). Similar paired analysis of fucose, bisecting GlcNAc, and sialic acid showed a small but statistically significant enhancement of binding affinity to FcγRII among otherwise matched but variably bisected variants when data from all FcγRII receptors and both experiments were considered (**Supplemental Figure 1**). However, this relationship was not observed to hold in both individual experimental replicates.

**Figure 5:**
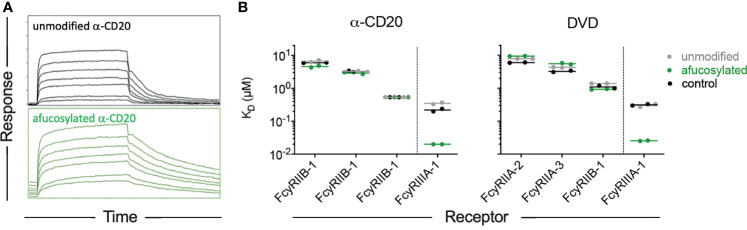
The fucose effect is generalizable across antibodies but not receptors. **(A)** Representative sensorgrams showing association and dissociation profiles of RM FcgRIIIa-3 from unmodified and afucosylated forms of a rhesusized version of rituximab. **(B)** Equilibrium binding affinities of CDR grafted CD20-specific antibodies with rhesus IgG1 Fc domains (left), and a dual variable domain (DVD) bispecific antibody (right) in unmodified and afucosylated forms, and in comparison to an unmodified rhesus IgG1 antibody of a differing specificity (control).

Because the effect of variable fucose content is of clinical relevance in both natural immune responses (19–24) and in optimization of antibody therapy (31, 53), we further probed this aspect of RM receptor binding profiles with additional antibody specificities. Rhesusized antibody to CD20 engineered to lack fucose (**Supplemental Figure 2**) showed improved binding affinity to RM FcγRIII, as did a Dual Variable Domain (DVD) format bispecific (**Figure 5**) that was similarly modified to reduce fucose content (**Supplemental Figure 3**). These experiments show that fucose effect is consistent across both rhesus and human IgG1 Fc domains in the context of distinct antibody specificities and even formats. Indeed, the slowed dissociation rate of afucosylated IgG Fc forms was apparent across data collected in this study, including the experiment for which that effect was obscured by concomitant changes to the association rate for FcγRIIIa-3 that appeared to affect the equilibrium affinities reported here. Importantly, the functional consequences of improved FcγRIII binding afforded by afucosylation are well established in mice and humans, where improvements in ADCC activity are readily observed to result [summarized in (58)]. While it appears that similar observations have yet to be reported in assays conducted with macaque effecter cells, whose receptor expression profiles are poorly defined and genetic diversity in receptors is extensive, the same glycosylation site associated with this phenotype is conserved in rhesus FcγRIII allotypes. The lack of this glycosylation site in other FcγR (e.g.: FcγRI and FcγRII) explains the receptor-specific nature of the “fucose effect”.

**Figure 6:**
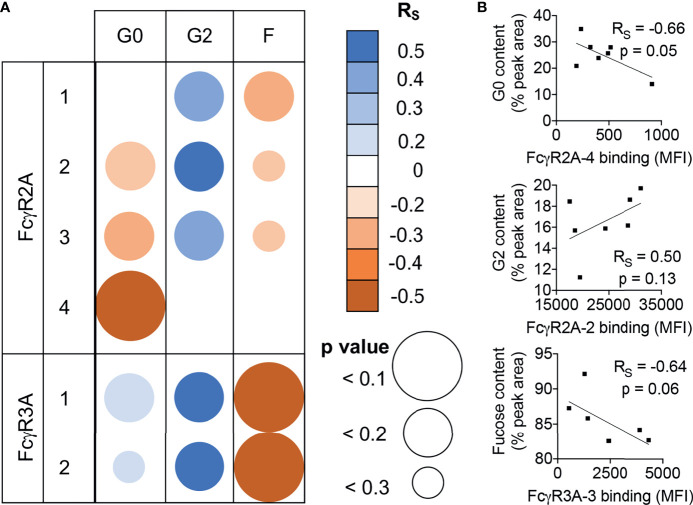
Similar FcγR binding glycopreferences are observed for RM serum IgG. **(A)** Correlations of receptor binding signal in multiplex assay and glycan species prevalence in rhesus macaque serum IgG for each allotypic variant of RM FcγRIIa and FcγRIIIa. Unadjusted Spearman correlation coefficient (R_S_) strength and direction are indicated in color, and confidence (p value) in size. **(B)** Exemplary scatter plots of relatively stronger correlative relationships between relative galactose and fucose content (% peak area) in relation to FcγR binding median fluorescent intensity (MFI). Correlation coefficients and exact p values are indicated in inset.

Lastly, to begin to further generalize these observations about glycan binding preferences beyond the human IgG1 backbone, we analyzed serum IgG glycoprevalences among a small set of healthy RM, and investigated relationships between galactose and fucose content and binding to RM FcγR. Though confidence in correlative relationships between glycan profiles and receptor binding signals are limited by small sample size and the potential effect of differing levels of serum IgG between animals, increased binding of serum samples with greater levels of Fc galactosylation was observed across diverse FcγRII and FcγRIII allotypes, as was the effect of reduced fucosylation for FcγRIII (**Figure 6**). Collectively, these observations support further generalization of the effects these glycoprofiles have on FcγR binding to polyclonal pools of mixed specificity, light chain usage, and subclasses of rhesus macaque IgG.

## Conclusions

This work establishes that the low affinity Fcγ receptors of RM demonstrate preferences for glycoforms of human IgG1 that largely mirror that of their human equivalents. Rhesus macaques therefore likely serve as an appropriate model for the evaluation of glycoengineered human antibodies. While the panel of glycovariants focused on human IgG1, this subclass is overwhelmingly represented among therapeutic monoclonal antibodies, and where evaluated, the glycan preferences appear to hold across human IgG subclasses (32, 59). We show here that the effect of fucose on RM FcγRIII recognition is generalizable across both human and rhesus monoclonal antibodies with distinct variable domains, a dual variable domain bispecific construct, and to polyclonal rhesus serum IgG. These results have important implications for the use of RM to study recombinant glycoengineered antibodies in preclinical and mechanistic studies of antibody therapies, as well as in attempting to relate antibody glycotypes raised in response to vaccination (60–62) or *via* vectored antibody delivery (63), to *in vitro* effector activities or *in vivo* outcomes.

## Methods

### Protein Expression and Purification

The engineering and characterization of the variably glycosylated panel of anti-TNP human IgG1s (50, 51) and rhesus macaque FcγRs (38) have been described previously. These modifications include manipulation of fucose (F), bisecting GlcNAc (B), sialic acid (S), and galactose (G) content (**Supplemental Table 1**). Unmodified anti-CD20 [anti-CD20 (2B8R1), Nonhuman Primate Reagent Resource Cat# PR-2287, RRID: AB_2716323] and afucosylated anti-CD20 [anti-CD20 (2B8R1F8), Nonhuman Primate Reagent Resource Cat# PR-8288, RRID: AB_2819341] were derived from rituximab and grafted into rhesus variable regions. Representative mass spectrometry-based glycoprofiles of these reagents are shown in **Supplemental Figure 2**. Unmodified and afucosylated forms of an HIV-specific double variable domain bispecific Ab that binds to both gp120 and gp41 of the envelope protein through fusion of CD4 (d2) with gp41-specific 7B2 monoclonal antibody linked through the H4 linker CD4 (d2)-H4-7B2 (64, 65) were constitutively expressed in either wild type or fucosyl-transferase Fut8-/- CHO cells. Unmodified and afucosylated DVDs showed equivalent binding to antigen by ELISA, but differential interaction with biotinylated fucose-specific *Lens culinaris* lectin, and the human CD16-expressing KHYG-1 Natural Killer cells (66) (kindly provided by Dr. David Evans, Wisconsin National Primate Research Center) (**Supplemental Figure 3**).

### Surface Plasmon Resonance

Antibodies were covalently coupled to a carboxymethyldextran-functionalized sensor (CMD200M, Xantec Bioanalytics) using carboiimide chemistry. A Continuous Flow Microspotter (CFM) (Carterra) allowed the complete panel, with replicates, to be printed on a single sensor chip. The sensor surface was activated by a mixture of 10.4 mM EDC (ThermoFisher, 77149) and 2.8 mM sulfo-N-hydroxysuccinimide (ThermoFisher, A39269) formulated in 10 mM MES (pH 5.0). Antibodies formulated in 10 mM sodium acetate (pH 5.0) at 50 and 100 nM were applied to the activated regions for 10 minutes. The regions were then washed with sodium acetate for 5 minutes. Unreacted substrate was capped using 1 M ethanolamine (Sigma-Aldrich, 15014-100ML) applied by the flow cell of the imaging-based surface plasmon resonance instrument (SPRi) (MX96, IBIS Technologies). Remaining ligand was removed and the overall capacity of the sensor tested using successive injections (5 rounds in total) of 25 μg/mL anti-human Fc and 10 mM glycine (pH 3.0).

The Fc receptor analytes were formulated at 20 μM in a running buffer consisting of 1x phosphate buffered saline containing 0.05% Tween 20. Each receptor was tested over an 8-point series of 1:3 dilutions, running from the lowest concentration to the highest. Association was measured over a 5-minute period before switching the flow cell to running buffer to capture 5 minutes of dissociation. Two blank injections of the running buffer following each receptor series were sufficient to completely dissociate these low-affinity analytes and prepare the sensor for the next receptor.

Raw data was processed using SprintX (IBIS Technologies). The background signal of the nearest unconjugated interspot was subtracted from the adjacent regions of interest to account for bulk shift and non-specific binding. The blank injection immediately preceding each series of receptor was also subtracted from each concentration of receptor. Equilibrium affinity values (K_D_) for each receptor-glycovariant pair were calculated in Scrubber 2 (BioLogic Software) using the average signal during a ten-second window at the end of the association phase when the system had reached equilibrium. The maximum response (Rmax) predicted for a saturated system was calculated for each region of interest. Regions with an Rmax of less than 25 were discarded for insufficient signal.

### Rhesus Serum IgG Receptor Binding and Glycan Analysis

Serum samples from seven healthy rhesus macaques were profiled for binding to rhesus FcγR-conjugated fluorescent beads in a multiplexed assay (67). Briefly, recombinant rhesus FcγR were covalently coupled to uniquely fluorescently coded magnetic microspheres, incubated in dilute serum, and bound antibody was detected with a phycoerythin-conjugated anti-IgG detection antibody prior to data acquisition on a Luminex FlexMap. Median fluorescent intensities were reported for each sample for each FcγR.

For glycan analysis, rhesus macaque IgG was purified from serum *via* Melon Gel (manufacturer), followed by digestion with both SpeB and IdeA enzymes (Genovis), and purification of cleaved Fc domains by Protein A affinity chromatography (GE Life Sciences), each according to the manufacturer’s recommendations. IgG glycan analysis was performed as described previously (68). Briefly, purified Fc was treated with PNGase F (New England Biolabs). Subsequently, protein was precipitated with ethanol and released glycans were evaporatively concentrated prior to fluorescent labeling with 2-aminobenzamide. After washing and removal of excess dye, glycans were analyzed using HILIC HPLC on a 150 3 2-mm TSKgel Amide-80 column (Tosoh Bioscience) with 3-mm packing material on a 1200 series HPLC (Agilent Technologies). Peak identities were confirmed *via* use of a glycan standard (Ludger). Quantification by area-under-the-curve analysis was performed with ChemStation software (Agilent Technologies).

### Statistical Analysis

Statistical analysis was performed in Graphpad Prism version 9. Global comparisons (**Figure 2**) of glycovariants with (+) and without (-) fucose, bisecting GlcNAc, and sialic acid modifications were evaluated for each individual receptor allotype by t test. Galactose content, which was alternatively increased, unmodified, or decreased, was evaluated by one-way ANOVA adjusted for multiple comparisons according to the procedure of Benjamini, Krieger, and Yekutieli. Paired comparisons (**Figure 4** and **Supplemental Figure 1**) of glycovariants with and without fucose, bisecting GlcNAc, and sialic acid modifications but for which other glycan modifications were held constant (i.e.: for fucose content, +G was paired with -F+G, and +G+S was paired with -F+G+S) were evaluated by paired t test across of FcγRII types and allotypes and FcγRIII allotypes. Paired comparisons evaluating the effect of variable galactosylation were evaluated using a mixed effect model with Tukey’s test of multiple comparisons, comparing the effect of each galactose characteristic (+G, unmodified G, and -G) when other modifications (F, B, and S)) were held constant. Strength and direction of relationships between Fc glycoform prevalences in rhesus serum IgG and FcγR binding signals were evaluated by Spearman’s rank correlation coefficient and statistic.

## Data Availability Statement

The raw data supporting the conclusions of this article will be made available by the authors, without undue reservation.

## Author Contributions

Conceptualization, AC and MA. Investigation, AC, GD, SP, WG, and NO-O. Writing – original draft, AC and MA. Writing – review and editing, all authors. Data analysis and curation, AC, NO-O, and MA. Funding acquisition, GV and MA. All authors contributed to the article and approved the submitted version.

## Funding

This work was supported in part by the NIGMS and the NIAID R01 AI131975, NIAID P01 AI120756, AI136758, and NIH NCI supplement to 2P30 CA 023108-41, the BioMT Molecular Tools Core supported by NIGMS COBRE award P20-GM113132. Reagents provided by the NIH Nonhuman Primate Reagent Resource were supported by the awards P40 OD028116 (ORIP) and U24 AI126683 (NIAID).

## Conflict of Interest

WG was employed by Antagen Pharmaceuticals Inc.

The remaining authors declare that the research was conducted in the absence of any commercial or financial relationships that could be construed as a potential conflict of interest.

## Publisher’s Note

All claims expressed in this article are solely those of the authors and do not necessarily represent those of their affiliated organizations, or those of the publisher, the editors and the reviewers. Any product that may be evaluated in this article, or claim that may be made by its manufacturer, is not guaranteed or endorsed by the publisher.
