# Supplementary material for: Biophysical Evaluation of Rhesus Macaque Fc Gamma Receptors Reveals Similar IgG Fc Glycoform Preferences to Human Receptors
Source: Front Immunol. 2021 Oct 12;12:754710. doi: 10.3389/fimmu.2021.754710 (PMC8546228; doi:10.3389/fimmu.2021.754710)
Supplement: Supplementary file 1 [file DataSheet_1.pdf]

Supplemental Figures	
Supplemental Figure 1	Paired comparison of the effect of glycan modification on Fc receptor binding affinities.
Supplemental Figure 2	Mass-spectrometry characterization of rhesus IgG1 mAb variants.
Supplemental Figure 3	Characterization of unmodified (wild type) and afucosylated DVD Abs.
**Supplementary Tables**	
Supplemental Table 1	Glycovariants analyzed in this study.

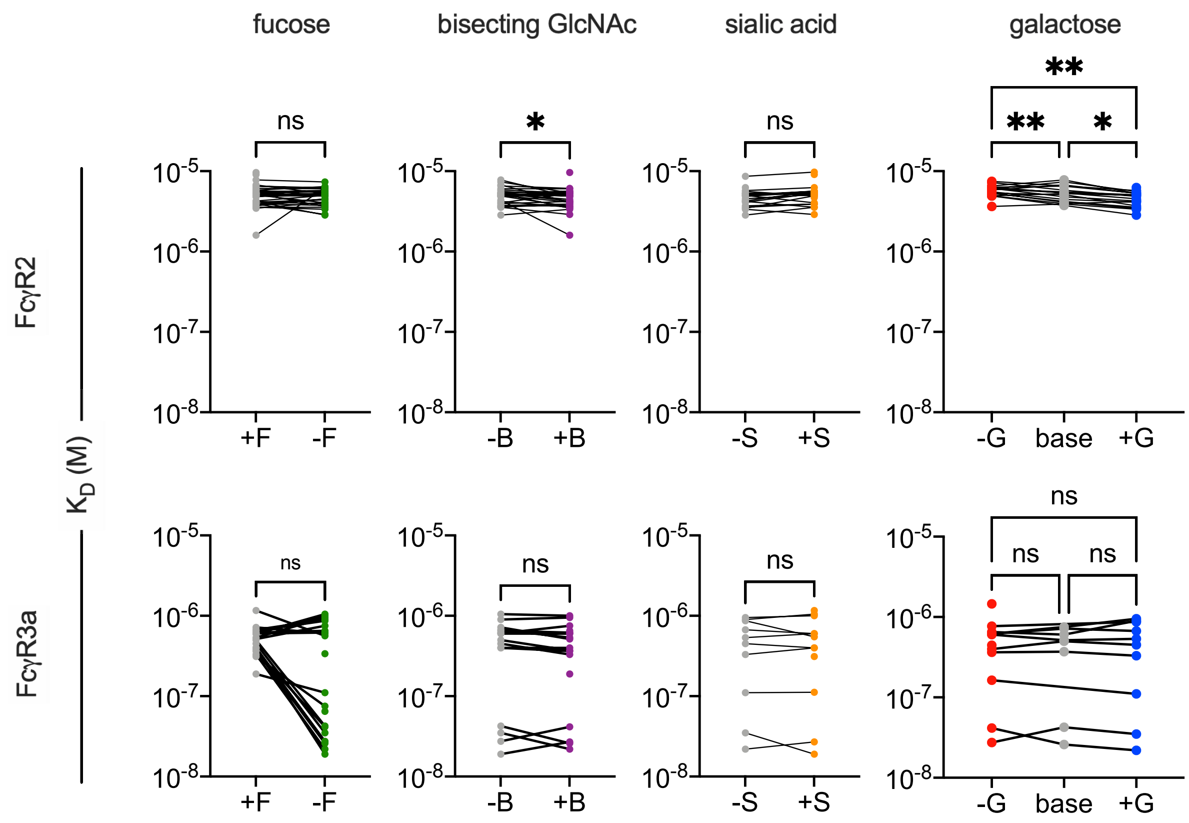

Supplemental Figure 1. **Paired comparison of the effect of glycan modification on Fc receptor binding affinities.** The effect of increased and decreased fucose, bisecting GlcNAc, sialic acid, and galactose content on binding to FcγR2 (top) and FcγR3 variants. IgG Fc glycotypes for which other glycan characteristics were held constant (e.g., differing fucose levels for preparations with matched levels of sialylation, bisection, and galactosylation). Results from both experimental runs are plotted. Statistically significant differences were tested by paired t test for fucose, bisecting GlcNAc, and sialic acid, and using a mixed effect model with Tukey's test of multiple comparisons for galactose (*$p<0.05$, **$p<0.01$, ***$p<0.001$).

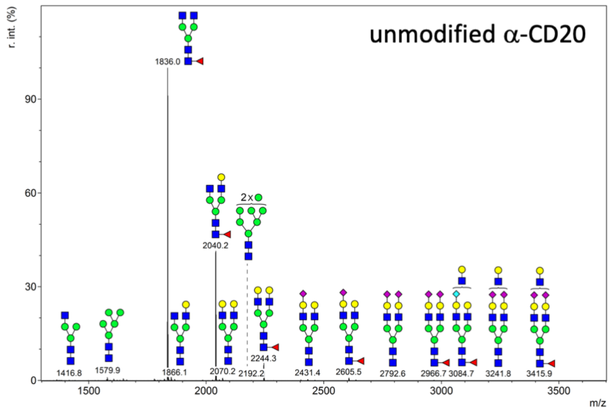

Supplemental Figure 2. **Mass-spectrometry characterization of rhesus IgG1 mAb variants.** N-glycan analyses confirm afucosylation of glycoengineered antibodies. The relative prevalence, of N-glycan structures as defined by peak intensity was determined by mass spectrometry. Symbols represent monosaccharides (hexNAc - blue square, mannose - green circle, galactose - yellow circle, sialic acid – purple diamond, fucose – red triangle). As expected, the most common glycan structures of glycoengineered antibody contained no fucose.

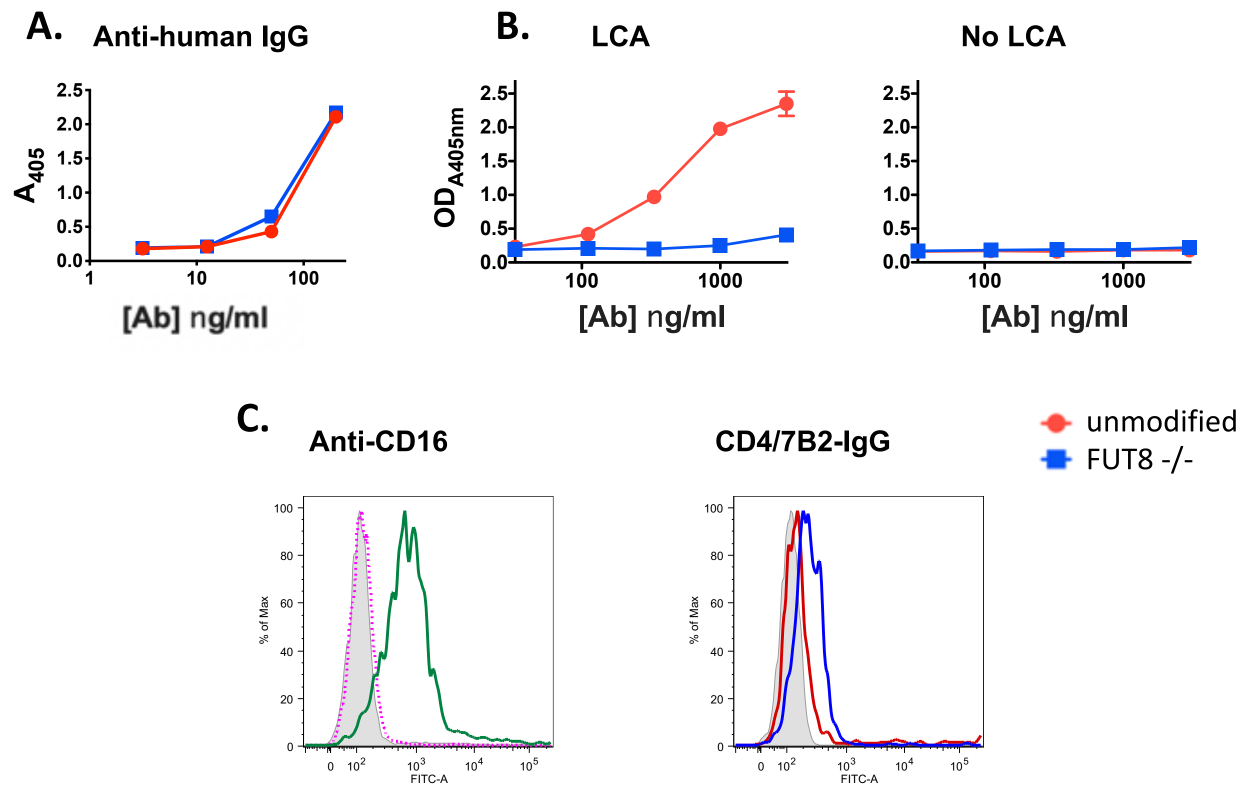

Supplemental Figure 3. Characterization of unmodified (wild type) and afucosylated DVD Abs. In all panels the wild type is shown in red, and the afucosylated form expressed in FUT8 -/- knockout cells in blue. **A**. ELISA demonstrating binding to gp41 peptide. Plates were coated with a peptide representing the epitope of the 7B2 variable domain, washed and detected with alkaline phosphatase conjugated anti-human IgG and the colorimetric substrate p-nitrophenyl phosphate (Sigma). **B**. Lens culinaris agglutinin binds DVD expressed from unmodified cells, but not the afucosylated form expressed from FUT8 -/- knockout cells. Plates were coated with the indicated concentration of Ab. Biotinylated Lens culinaris agglutinin (Vector Labs) 1µg/mL (left) or 0 (right) was added, followed by alkaline phosphatase avidin, and substrate. **C**. Binding of DVD to huCD16+ KHYG-1 NK cells by flow cytometry. Left shows the expression of CD16 on the cell surface. Isotype control is shown in gray, anti-CD16 in green. The right-hand panel shows binding of the DVDs to the CD16+ cells. Ab (10 µg/mL) was incubated with the cells for 1 hr, washed, and detected with fluoresceinated anti-human IgG.

Supplemental Table 1. Glycovariants analyzed in this study.

Glycomodification	Fucose	Bisecting GlcNAc	Galactose		Sialic acid
	reduction	addition	reduction	addition	addition
unmodified					
-F					
-F+B					
-F+B-G					
-F+B+G					
-F+B+G+S					
-F-G					
-F+G					
-F+G+S					
+B					
+B-G					
+B+G					
+B+G+S					
-G					
+G					
+G+S					
